# 3D Considerations and Outcomes of Immediate Single Implant Insertion and Provisionalization at the Maxillary Esthetic Zone: A Long-Term Retrospective Follow-Up Study of Up to 18 Years

**DOI:** 10.3390/jcm10184138

**Published:** 2021-09-14

**Authors:** Eitan Mijiritsky, Antonio Barone, Ihsan Caglar Cinar, Katalin Nagy, Maayan Shacham

**Affiliations:** 1 Tel-Aviv Sourasky Medical Center, Department of Otolaryngology, Head and Neck and Maxillofacial Surgery, Tel Aviv-Yafo 6139001, Israel; 2 The Maurice and Gabriela Goldschleger School of Dental Medicine, Tel Aviv University, Tel Aviv-Yafo 6139001, Israel; 3 Unit of Oral Surgery, Department of Surgical, Medical, Molecular Pathologies, and Critical Needs, School of Dental Medicine, University of Pisa, 56128 Pisa, Italy; barosurg@gmail.com; 4 Department of Oral Implantology, Faculty of Dentistry, Istanbul University, 34093 Istanbul, Turkey; cinarcaglar@gmail.com; 5 Department of Oral Surgery, University of Szeged, Tisza L. krt 64, 6720 Szeged, Hungary; katalin.nagy@universityszeged.com; 6 School of Social Work, Ariel University, Ariel 40700, Israel; Drmaayanshacham@gmail.com

**Keywords:** prosthodontics, soft tissue-implant interactions, bone implant interactions, long-term, retrospective

## Abstract

Aim: Long-term studies addressing the outcomes of single immediate implantation and provisionalization at the maxillary esthetic zone are needed. The current study aimed to assess such outcomes along a follow-up period of up to 18 years. Materials and methods: The current study is a continuation follow-up of our previously published up to 6-year follow-up study, dated between the years 2002–2008, performed in a private clinical practice in Tel-Aviv, Israel. A total of 15 patients (23 implants) who had been treated for single-tooth replacement at the maxillary esthetic zone since 2002, underwent clinical and radiographic follow-up evaluations. Primary outcomes included mean Marginal Bone Levels (MBL), with Bleeding on Probing (BOP), implant success rate, prosthetic and esthetic complications evaluated as secondary outcomes. Results: The implant success rate was at 100%. Bone remodeling processes were observed over the follow-up period, with 0.9 mm mean marginal bone loss observed during the first 6 years of observation, followed by −0.13 ± 0.06 mm mean loss after 6 to 18 years. The last finding suggests bone deposition, as reported by other studies (Donati et al., 2012). At the final radiographic evaluation, a mean MBL of 1.35 mm ± 0.16 was demonstrated. No differences with respect to implant type or site were found. A generalized absence of BOP and esthetic complications occurred in two cases as a result of continuous adjacent teeth eruption versus obvious implant ankylosis. Conclusions: Adhering to careful clinical protocols and 3D bone to implant considerations while immediately placing an anterior implant, this treatment approach offers both stable and esthetically acceptable results for the replacement of missing teeth at the maxillary esthetic zone.

## 1. Introduction

Immediate implant insertion and provisionalization at the maxillary esthetic zone is considered to be a viable treatment option for those with missing teeth in that area [1,2,3,4,5,6]. Successful results of immediate implant insertion and provisionalization in the anterior maxilla were first documented by Wöhrle [7], with many studies following since [2,3,4,5,6,8,9,10,11,12,13,14,15,16,17,18]. Immediate implant insertion and provisionalization procedures have many advantages, one of which is the ability to preserve gingival and bony architecture [7,19,20,21]. Other advantages may include shorter treatment duration, reduced costs, improved patient comfort, and immediate esthetic results [19,22]. Achieving esthetic success is suggested to be dependent on an ideal three-dimensional implant position, in the apico-coronal, anterior-posterior, and mesio-distal dimensions, allowing the maintenance of adequate bone volume and consequently soft tissues volume surrounding the implant surface [23,24,25,26,27,28].

There might be several disadvantages of immediate loading such as micromotion and implant instability [29,30], which may inevitably deteriorate to implant loss. Successful cases may be influenced by both patient and clinician-dependent variables. Proper diagnosis, treatment planning, and careful case selection play a crucial role in order to obtain the desired and favorable esthetic outcome [19].

Marginal bone levels (MBL) after immediate implant insertion and provisionalization seem to be equally successful to those levels after early and conventional implant loading, as described by recent systematic reviews and meta-analyses [25,26]. It may be assumed that a mean MBL may be reduced by 1.0 ± 0.6 mm from initial implant placement to 18 months thereafter for implants restored using the conventional approach [22]. In our previously published up to 6-year follow-up study [2], a mean MBL loss of 0.9 ± 1.1 mm was described, which is similar to those described by other studies [22,27,28]. Soft tissue aspects of immediate implant placement and provisionalization, including but not limited to bleeding on probing (BOP), seem to be of equal quality to conventionally loaded implants [22,25,29]. Data regarding implant success rates differences between immediate and conventional placement seem to be controversial, as immediately-loaded implant success rates may be lower [25] or similar [26] to conventionally-loaded implants.

As most existing data regarding immediate implant placement and provisionalization deal with short-term follow-ups, as described by several systematic reviews and meta-analyses, for example, [25], there is a need for long-term studies (>5 years follow-up) in order to better evaluate the consequences of such procedures. In addition, there is a limited number of studies regarding immediate loaded single implants in the anterior maxilla, with limited knowledge regarding its effect on peri-implant MBL and soft tissue responses [22]. A recent long-term (5–10 years follow-up) retrospective study on implants placed in an immediate provisionalization protocol [30] reported high survival rates and stable MBL among immediate implants with provisionalization, with an additional study (1–8 years follow-up) reporting improved hard and soft tissue outcomes using such approach [23].

Therefore, the aim of the current retrospective study is to evaluate the long-term follow-up period of up to 18 years of immediately placed and provisionalized implants in fresh-extraction sites at the maxillary esthetic zone, in terms of marginal bone levels, bleeding on probing, implant success rates, prosthetic and esthetic complications.

## 2. Materials and Methods

### 2.1. Patients

The current study is a continuation follow-up of our previously published up to 6-year follow-up study, dated between the years 2002–2008, performed in a private clinical practice in Tel-Aviv, Israel [2], and conforms to the recognized standard as stated in the Declaration of Helsinki. The study conforms to the STROBE guidelines and has gained the Ethical Approval of the Ethics Committee of Tel-Aviv University, Israel.

In this retrospective study, 15 patients (7 female and 8 male) ranging in age from 23 to 62 years, who were treated for single-tooth replacement at the maxillary esthetic zone since 2002, were selected for clinical and radiographical follow up evaluations, with a total of 23 implants. Following a thorough explanation of the treatment alternatives as well as risks, those accepting replacement by immediate loading and provisional crown placement were included. Informed consent was obtained from all patients before the clinical procedure.

The current study addresses clinical and radiographical evaluations between the years 2002–2020 of fifteen patients with 23 implants, as one of the patients was considered as ‘drop-out’ as he did not show for follow-ups due to unknown reasons.

### 2.2. Pretreatment Examination

The oral examination was focused on stable bilateral occlusion, soft tissue condition, buccolingual and mesiodistal width of soft and hard tissues and intermaxillary relationship. Periapical radiographs using the parallel technique, panoramic radiographs and computerized tomograms were also obtained, as necessary.

Inclusion criteria for tooth procedure of extraction and immediate implant placement and provisionalization included:

1. Older than 18 years of age;
2. Tooth loss due to periodontal attachment loss,
3. Non-restorable crowns,
4. Endodontic failures,
5. Root fracture,
6. Dentoalveolar trauma.

Exclusion criteria for this clinical procedure included:

1. Uncontrolled diabetes;
2. Severe para-functional habits (bruxism or clenching);
3. Infected adjacent teeth;
4. The need for tissue augmentation procedures during surgery.

### 2.3. Surgical Technique

Implant placement and immediate provisionalization were performed by a single experienced clinician (E.M.). All patients were given Amoxicillin 1 gr. 1 h prior to surgery. Gentle elevation of the tooth root was performed to preserve the alveolar housing around the extraction site. Flaps were avoided. Atraumatic extraction was done using a periotome to release the periodontal ligament. Once the tooth was removed, the socket was carefully debrided and irrigated with sterile saline. Tapered titanium implants were placed (Xive and Frialit-2, Dentsply/Friadent, Mannheim, Germany and Seven, MIS Implant Technologies Ltd., Bar-Lev Industrial Park, Misgav, Israel).

Implants with diameters of 3.3 to 5.5 and lengths of 13 to 16 mm were selected based on the size of the tooth socket and mesiodistal diameter of the tooth to be replaced.

Implants were placed according to an ideal three-dimensional bone to implant position, in the apico-coronal, anterior-posterior, and mesio-distal dimensions.

The platform of the implant was set approximately 1.5–2 mm below the level of the buccal bone. Implant placement respected the minimal 1.5–2 mm space between the adjacent tooth and the implant. Implants were positioned palatally in relation to the natural ‘central’ position where the tooth should have been, and an autogenous bone graft obtained by using a drill was used to fill space discrepancies in the cervical area when gaps were 2 mm or greater.

Implant stability was monitored by using a manual torque wrench and the insertion torque was recorded in N/cm. If insertion torque values were 32 N/cm or greater, the implants were included in the study.

Following placement, each implant was connected to a prefabricated plastic provisional abutment, as previously described [2,31]. The fixed provisional restorations were cemented to the abutments (Temp Bond, Brea, CA, USA). Cement removal was carefully performed using scalers and floss. Occlusal contacts were avoided using polyester film and articulating paper permitting immediate but reduced functional loading of the implants.

Patients were asked to limit their diet to soft food for one month and were routinely examined once a week for 3 weeks and then once a month for 6 months.

### 2.4. Follow-Up and Definition of Outcome Variables

The primary outcome parameter of this retrospective study was the measurement of MBL. The marginal bone level was recorded at the mesial and distal aspect of each implant after provisionalization (baseline) and during follow-ups up to 18 years. The distance between the implant-abutment interface to the first bone-to-implant contact (so-called bone level) was assessed on periapical radiographs obtained using a positioning device and the long-cone parallel technique at baseline and at follow-ups. The implant length served as the reference distance during these measurements. The marginal bone level was calculated at follow-ups by subtracting bone levels at these years from baseline bone levels. Mesial and distal values were averaged in order to obtain a single value per implant.

The secondary outcome parameters were the BOP, implant success, and prosthetic complications. BOP was assessed at six locations (mesiobuccal, buccal, distobuccal, mesiopalatal, distopalatal, and palatal) around the implant at follow-ups. Each location was scored 0 or 1 (absence or presence of bleeding on probing, respectively). Implant success was defined as the mere presence of the implant irrespective of other clinical parameters. Prosthetic and esthetic complications were recorded as well. All measurements of peri-implant tissue responses were done by a single examiner (E.M). Statistical analyses were conducted utilizing SPSS Statistics version 26 (IBM, Armonk, NY, USA).

## 3. Results

Table 1 presents an overview of the clinical data of the patients and implants included in this study. A total of 23 implants were placed at the maxillary esthetic zone in 15 patients—7 women and 8 men with an average age of 39.9 years ranging from 23 to 62 years. Follow-ups starting at implantation day ranged from 168 months (14 years) to 216 months (18 years) with a mean follow-up of 187.37 months (15.99 years).

The mean MBL around the remaining implants, as measured in the last follow-up radiograph was at 1.35 mm ± 0.16. The pre and post-operative radiographs compared with the follow-up radiographs showed a mean marginal bone loss from placement to the 6-year follow-up, from placement to last follow-up, and between the 6-year follow-up to the last follow up time point, as described in Table 2. No differences were found with respect to abutment type or site. Overall implant success rates were at 100%. There was a generalized absence of bleeding on probing. There were two cases of esthetic complications as a result of continuous adjacent teeth eruption accompanied with implant ankylosis. These esthetic concerns were managed using solely prosthetic procedures, that is, definitive restoration replacement.

## 4. Discussion

In the current retrospective clinical follow-up study of up to 18 years, a 100% survival rate was observed among immediately placed and provisionalized implants in the esthetic zone of the maxilla. This high rate coincides with other long-term follow-up studies using immediate placement and loading protocols at the esthetic zone [32,33,34,35,36]. Based on the same study sample, the current study is a continuation of a previous follow-up study [2]. The current study includes 23 implants, as opposed to the 24 implants included in the aforementioned study, due to a single patient (and implant) dropout.

The mean marginal bone loss in immediate implants with the non-occluding provisional crown during the first follow-up period of up to 6 years was found at 0.9 mm, as previously described [2]. In the current long-term follow-up study, a mean marginal bone loss of 0.79 ± 0.5 mm from baseline to follow-up and mean marginal bone loss of −0.13 ± 0.06 mm between the 6–18 years follow-up periods are reported. As can be noted, there was a decrease in MBL between the follow-up periods, indicating bone deposition. This finding was also found in a 5-year follow-up study by Donati et al. [33] who also observed bone gain in immediate placed and loaded implants at the esthetic zones of both maxilla and mandible, with ~70% of implants showing no bone loss. However, no information was provided regarding whether the provisional restorations were in occlusion. Van Nimwegen et al. [37] also reported a mean marginal bone loss of 0.31 ± 0.2 mm after a mean follow-up of 4 years (up to 7-year follow-up) in immediate implant insertion and non-occluding provisionalization at the esthetic zones of both jaws, with 0.06 ± 0.1 mm between definitive crown placement to follow up. These findings may be also supported by the success criteria defined by Albrektsson and Zarb [38] and used by Degidi et al. [36] in their study on 111 immediately, non-occluding single implants place at both jaws’ esthetic zones, providing <0.2 mm of marginal bone loss after the first year as a success criterion. The study reported a mean marginal bone loss of 0.9 ± 0.2 mm after a 5-year follow-up, which coincides with our results.

In the initial healing period following immediate provisionalization of implants in the esthetic area, there may be an accelerated bone resorption period, as suggested by Donati et al. [33], and was observed in our initial follow-up period of up to 6 years [2]. Lang et al. [34] reported a mean marginal bone loss of 0.70 ± 0.26 mm at a 5-year follow-up of tapered implants placed in esthetic zones of both mandible and maxilla, using the immediate non-occluding provisionalization approach. This agrees with the mean marginal bone loss over the 18-year follow-up described in our study. As can be seen, there is currently a limited number of long-term (>10 years) follow-up studies on immediate implantation and provisionalization of implants placed at the maxillary esthetic zone. To the best of our knowledge, the current study has the longest follow-up period (14–18 years) of immediate implantation and provisionalization of implants placed at the maxillary esthetic zone, up to the present date. Long-term follow-up studies are crucial in order to better evaluate the impact of such clinical procedures on the human body tissues [39]. Therefore, in a careful evaluation, it may be assumed that after 18 years of clinical function, implants that were immediately inserted and provisionalized in a non-occlusion manner maintain stable marginal bone levels, as performed by the conventional approach [33].

In the current study, BOP served as the marker for gingival inflammation. Donati et al. [33] reported an increase of BOP measured at four sites (mesial, distal, buccal, and palatal) between 1-year to 5-year follow-ups, with proximal peri-implant sites showing higher frequencies, and did not find statistically significant differences between conventional and immediate loading protocols. The study also found a positive correlation between positive BOP cases and marginal bone loss. In a long-term retrospective follow-up (5–10 years) study on non-occluding immediately placed implants in both mandible and maxilla, Maló et al. [32] measured BOP and presented the results among other variables under the umbrella term ‘peri-implant pathology’, affecting 11.6% of investigated implants. On the other hand, in the current study, there was a generalized absence of BOP. This may be due to proper oral hygiene maintenance and oral hygiene maintenance instructions were given after initial implant insertion and provisionalization and at each follow-up session, as suggested by Maló et al. [32]. Our findings are in agreement with Van Nimwegen et al. [37] and Lang et al. [34], who, by using the bleeding index and the Modified Gingival Index, respectively, did not find a statistically significant difference in gingival health between baseline and the 5-year follow-up.

**Figure 1:**
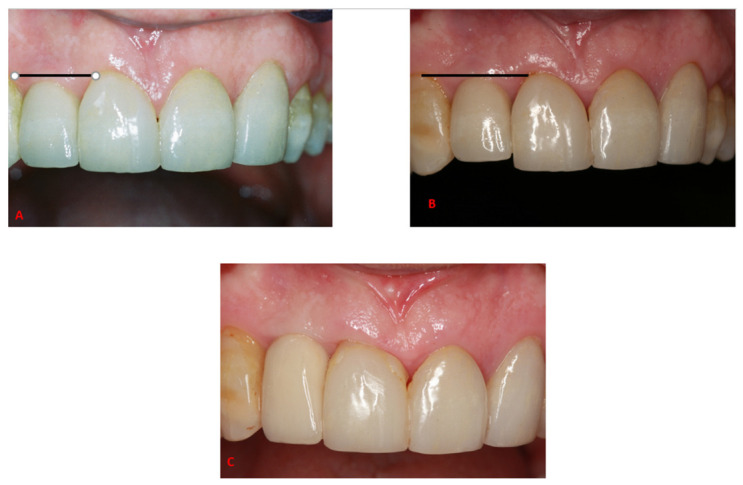
A 59-year-old male patient, 10 years after the placement of a right lateral incisor implant. It can be noticed when comparing the picture from 2003 (**A**) to the one from 2013 (**B**), the submerged position of the crown retained implant of the right lateral incisor in 2013, in comparison to the right central incisor, due to a continuous adjacent tooth eruption, leading to a short and submerged crown and a non-anatomic gingival line and unesthetic incisal line (**B**). (**C**) shows the result after the replacement of the final restoration.

Although absent in our current long-term follow-up study, prosthetic complications may arise over long-term follow-up of immediate implantation and provisionalization protocols. Maló et al. [32] reported a rate of 19.5% prosthetic complications, with 18.3% occurring during the first 5 years of follow-up and were related to the provisional stage, and 1.4% occurred beyond the 5-year follow-up. Donati et al. [33] reported three cases of technical complications among the 159 placed implants, all occurring along the first 5 years of follow-up. Thus, it may seem that once adjusted properly during the first years of functioning, prosthetic complications are rare to occur after 5 years of clinical service. Nonetheless, an esthetic complication of a continuous tooth eruption adjacent to single-implant restorations in the anterior maxilla was documented in two of the patients included within the current study sample (see Figure 1), as was reported in other studies as well [40,41,42]. As suggested, it may be that continuous facial skeleton growth and teeth eruption occur during the second to fifth decades of life. Therefore it may be advisable to delay placement of an anterior maxillary implant in the adolescent patient, inform the adult patient about this phenomenon, and to receive written informed consent before considering placing an anterior maxillary implant.

### Figure Legends

Following immediate placement and provisionalization of anterior maxillary single implants, a continuing gingival recession of the facial gingival tissue may also occur, along with possible spontaneous papilla regeneration over time, as suggested by a recent long term follow up (2–8 years) study [43], and supported by other systematic reviews [44]. Nonetheless, a recent systematic review renders it difficult to draw clear conclusions regarding such recession [45]. In addition, a recent RCT trial suggests that immediate provisionalization of implants placed at the maxillary esthetic zone fails to provide additional aesthetic results and that perhaps implant positioning is the most crucial for determining the facial mucosal level [46]. In the case of mucogingival recessions at the maxillary esthetic zone, immediate implant insertion and provisionalization was seen to significantly reduce initial mucogingival recession in a long-term (2–8 years) follow-up study [47].

Dual or co-axis subcrestal angle correction implants with variable platform switching (SAC/VPS) have recently been shown to increase peri-implant soft tissue thickness and reduce gingival recession in case of immediate implant placement and provisionalization at the esthetic maxillary zone [48]. It seems that aesthetic complications vary among different studies and further studies on this topic are warranted.

To sum up, for soft and hard tissue preservation, both conventional implant loading protocols and immediate implant placement and provisionalization at the esthetic maxillary zone may yield similar beneficial results, as found by randomized controlled trials, systematic reviews, and meta-analyses [22,33,45,49,50,51].

Presently, 3D preoperative planning through cone-beam computed tomography analysis (CBCT) allows for the verification of the maxillary anatomy and qualitative and quantitative bone structures before dental implant placement [52,53]. The CBCT Standard Triangulation Language (STL) files elaborated through specific surgical software, together with stereolithographic (SL) models, promote individual virtual planning [54] that allows immediate prosthetic loading of the implants with a reduction in surgical times, increasing the comfort of the patients [55,56,57] and the predictability of surgical results [58,59], especially as regards aesthetic aspects [27].

Guided implant surgery allows the transfer of planned rehabilitation projects directly into the surgical field hence it should be considered for adoption especially for these highly technique-sensitive surgical procedures in the esthetic zone of the anterior maxilla.

The current study has several limitations, including a relatively small number of patients and implants as part of its design. On the other hand, the benefit of such study design could be the ability to maintain a steady longitudinal long-term follow-up study (up to 18 years follow up) as dropouts are quite common in large scale studies, varying between 10–54.7% dropouts, for example, in [32,33,34,46,60,61]. In future studies, it may be beneficial to include a larger number of participants in a prospective, long-term, longitudinal randomized clinical trial setting. As stated, there is a lack of long follow-up studies on immediate implant placement and non-occluding provisional restoration at the esthetic maxillary zone, thus further studies in this topic are warranted.

## 5. Conclusions

In conclusion, in a selected group of patients with very good oral practices and oral hygiene maintenance, immediate implant insertion and non-occluding provisionalization aid in the preservation of soft and hard tissue architecture at the esthetic maxilla zone. Proper case diagnosis utilizing 3D analysis, clinical and radiographic measures, as well as careful patient selection, are of paramount importance to promote success. Altogether, this treatment approach serves as a viable treatment option for those with missing teeth in this area, with acceptable esthetics, long-term survival rate, and marginal bone loss over a long-term follow-up period (14–18 years).

## Figures and Tables

**Table 1:** Summary of basic demographic, site of implantation, implant type, diameter and length, and follow-up period.

Patient No.	Gender *	Age	Site	Implant *	Diameter	Length	Follow Up (Months)
1	2	62	12	1	4.5	15	201
2	1	55	12	2	3.4	15	180
3	1	61	22	2	3.8	15	216
		61	11	2	5.5	15	208
4	2	47	12	2	3.8	15	209
5	2	50	12	1	3.75	16	179
		50	22	1	3.75	16	“
6	2	23	11	2	4.2	16	194
		23	12	2	3.3	16	“
7	1	57	21	1	4.7	13	190
	1	57	11	1	4.7	13	“
8	2	52	24	3	3.8	13	174
9	1	25	12	1	3.75	13	174
		25	22	1	3.75	13	“
10	2	25	25	3	3.8	13	171
		25	24	3	3.4	15	“
11	1	32	12	3	3.8	15	179
		32	22	3	3.8	15	“
12	2	50	13	2	3.3	13	193
13	1	26	22	2	3.75	16	192
14	1	26	13	3	3.8	15	168
15	1	26	23	3	4.5	15	“
	s	28	21	3	4.5	15	170

* Implants: 1—Seven—MIS Implant Technologies Ltd., Bar-Lev Industrial Park, Israel; 2—Frialit 2—Friadent, Manheim, Germany; 3—XIVE—Friadent, Manheim, Germany. * Gender: 1—male. 2—female.

**Table 2:** Marginal Bone loss of immediate implantation and provisionalization of implants placed at the maxillary esthetic zone from base.

	Bone Loss Up to 6 Years (from Baseline)	Bone Loss between 6–18 Follow-Up Years (between Follow-Ups)	Bone Loss Up to 18 Years (from Baseline)
Mean (in mm)	0.9	−0.13	0.79
Std. (in mm)	0.5	0.06	0.5
Number of implants	24	23	23
